# Bibliometric analysis on the literature of monk fruit extract and mogrosides as sweeteners

**DOI:** 10.3389/fnut.2023.1253255

**Published:** 2023-08-29

**Authors:** Andy Wai Kan Yeung

**Affiliations:** Oral and Maxillofacial Radiology, Applied Oral Sciences and Community Dental Care, Faculty of Dentistry, The University of Hong Kong, Hong Kong, Hong Kong SAR, China

**Keywords:** non-nutritive sweetener, mogroside, monk fruit, bibliometric, RPYS, VOSviewer

## Abstract

The evolution of research literature on monk fruit extract and mogroside as sweeteners has yet to be investigated. No study has evaluated this literature from a bibliometric perspective. This bibliometric study analyzed the relevant research literature indexed in Web of Science, to unveil its growth and the most productive authors, institutions, countries, journals, and journal categories. In addition, this study aimed to identify the recurring themes of the literature. On July 2023, the Web of Science Core Collection database was accessed with the following search query: TS = (*mogroside* OR “luo han guo” OR “lo han kuo” OR “monk fruit*” OR “monkfruit*” OR “Siraitia grosvenorii”) AND TS = (sweet*). The search identified publications mentioning these terms in their title, abstract, or keywords. Only articles and reviews were included. No additional filters were placed on publication year, language, etc. Basic publication and citation frequency counts were recorded directly from the database. The complete record of the publications were exported into VOSviewer and CRExplorer, for visualization of recurring terms and identification of commonly cited references, respectively. The search yielded 155 publications. Publication and citation counts have increased steadily since the 2010s. The most productive authors and institutions were mostly based in Asian countries, such as China, Japan, and Singapore. Nearly half of the publications had contributions from China and were published in journals concerning food science technology. The health effects and biosynthesis of mogrosides were the recurring themes among the top 10 most cited publications. Most of the health effects, such as anti-hyperglycemic, anti-hyperlipidemic, and anti-diabetic properties, were demonstrated in animal models with limited evidence from clinical trials. Future studies should focus on testing in humans. Since monk fruit extracts were generally recognized as safe (GRAS) according to the Food and Drug Administration (FDA), the affirmation of these health benefits in humans by future studies should advocate its use in the food industry and the society to generally improve the public health.

## Introduction

Monk fruit extract is a natural sweetener derived from the fruit of the monk fruit tree (*Siraitia grosvenorii*), also known as “Luo Han Guo” or “Lo Han Kuo” in some papers. It is believed that the monk fruit has been cultivated in massive scale exclusively in the southern part of China, mostly in the mountainous areas of Guilin of the Guangxi Province (1, 2). After filtering out sugars such as fructose and glucose, monk fruit extract on the market is a non-nutritive sweetener that has gained popularity recently due to its sweetness and potential health benefits. This is especially relevant, as nowadays health authorities, food engineers, scientists, and health insurers advocated that consumers should maintain a balanced diet to sustain human health and prevent illnesses (3).

The sweetness of monk fruit extract comes from its high concentration of mogrosides, a group of glycoside of cucurbitane derivatives found in the fruit (4). Many of these compounds are much sweeter than sugar but do not contain any calories. For instance, mogroside V is the major component with approximately 250 times of sweetness compared to sucrose (5). Meanwhile, mogroside IV has a similar sweetness intensity as mogroside V, and mogrosides I and II have a similar sweetness intensity as sucrose (4). Mogroside is generally recognized as safe (GRAS) according to the Food and Drug Administration (FDA) of the United States (5). This makes monk fruit extract a good alternative to table sugar for people who wish to reduce their calorie intake.

Monk fruit extract is also a potentially healthy sweetener for patients with diabetes. Since the human body does not recognize mogrosides as carbohydrates or sugars, they do not trigger an insulin response. In a rat study, it was reported that mogroside V could improve blood glucose, increased glycogen synthesis, and alleviated insulin resistance in rats with type 2 diabetes (6). In addition to its sweet taste, monk fruit extract is also believed to have several health benefits. *In vitro* and *in vivo* studies have shown that the mogrosides may have antioxidant (7) and anti-inflammatory properties (8).

However, monk fruit extract is similar to other natural sweeteners such as stevia in providing bitter taste and metallic taste apart from sweetness, though it was reported that the such unpleased tastes seemed to be less intense for the former compared to the latter (9–11).

In brief, monk fruit extract and mogrosides are natural sweeteners that potentially offer a range of health benefits and are a popular alternative to sugar. They have high sweet intensity yet being non-nutritive. As more people prefer healthier alternatives to sugar, monk fruit extract may become more and more popular in the future. With this background, the literature on monk fruit extract and mogrosides as sweeteners was analyzed in bibliometric way, to reveal if they indeed received international attention in terms of research, and identify the recurring themes within this literature set.

## Methods

### Literature search and study selection

In July 2023, the electronic Web of Science (WoS) platform was accessed, and its Core Collection database was queried with the following search string: (*mogroside* OR “luo han guo” OR “lo han kuo” OR “monk fruit*” OR “monkfruit*” OR “Siraitia grosvenorii”) AND (sweet*) (Table 1). The term sweet* was added to focus the search on papers that considered the sweet taste. The search yielded 155 papers from WoS: 134 original articles and 21 reviews.

**Table 1:** Search strategy in Web of Science.

Domain	Details
Databases	Science Citation Index Expanded (1970–present) Social Sciences Citation Index (1956–present) Arts & Humanities Citation Index (1975–present) Conference Proceedings Citation Index – Science (2009–present) Conference Proceedings Citation Index – Social Science & Humanities (2009- present) Emerging Sources Citation Index (2005–present)
Search string	TOPIC = (*mogroside* OR “luo han guo” OR “lo han kuo” OR “monk fruit*” OR “monkfruit*” OR “Siraitia grosvenorii”) AND TOPIC = (sweet*)
Timespan	All years (1956–2023)
Document types	Articles and reviews only

The publication and citation frequency counts were directly extracted from the WoS platform. The counts from England, Scotland, North Ireland, and Wales were combined to represent United Kingdom. The complete record of the publications were exported into VOSviewer (12), to generate a term map with default parameters. The term map visualized the recurring terms (*n* ≥ 3) from the title and abstract of the papers. Each term was denoted by a node. The node size represented the publication count, its color reflected the citations per publication (CPP), and the inter-node distance indicated their co-occurrence. The top 10 most cited publications were identified and the context of their citations (supporting, mentioning, or contrasting) were evaluated from another online database, Scite (13). Besides, complete record of the publications were also exported into CRExplorer (14), to identify the early seminal works (references) often cited by these papers. The default parameters were used to generate a “reference publication year spectroscopy” (RPYS), which was a figure that showed multiple waves along the timeline representing years the cited references had much more citations relative to preceding 2 and succeeding 2 years (i.e., deviation from 5-year median) (15–20). Since the institution of the author only subscribed WoS back to year 1956, by using cited reference analysis here the author might be able to reveal important papers published before 1956.

Ethical approval was not applicable to this study.

## Results

**Figure 1:**
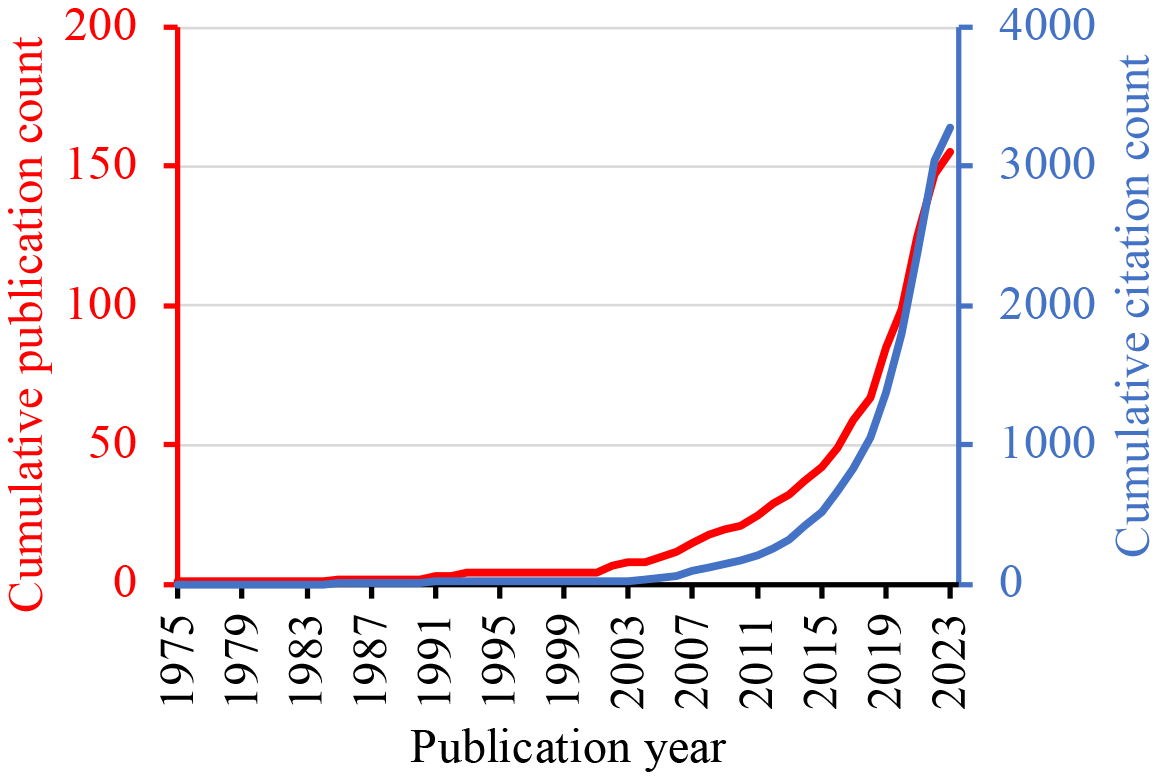
The cumulative publication (red line) and citation (blue line) counts of the literature on monk fruit extract and mogrosides as sweeteners.

Figure 1 shows the cumulative publication and citation counts of the literature on monk fruit extract and mogrosides as sweeteners. Both counts have increased steadily since the 2010s. Table 2 lists the top 5 most productive authors, institutions, countries, journals, and journal categories. The most productive authors and institutions were mostly based in Asian countries, such as China, Japan, and Singapore. Nearly half of the publications had contributions from China and were published in journals concerning food science technology.

**Table 2:** The top 5 most productive authors, institutions, countries, journals, and journal categories.

The most productive entities	Number of publications (% of 155)	Citations per publication (CPP)
**Author**		
Ma, Xiaojun	9 (5.8)	19.2
Luo, Zuliang	7 (4.5)	6.0
Murata, Yuji	6 (3.9)	44.0
Sun, Yuanxia	6 (3.9)	22.2
Li, Dianpeng	5 (3.2)	15.8
Sugiura, Masaki	5 (3.2)	50.4
**Institution**		
Chinese Academy of Sciences	17 (11.0)	18.4
Chinese Academy of Medical Sciences & Peking Union Medical College	10 (6.5)	31.9
Institute of Medicinal Plant Development, Chinese Academy of Medical Sciences	10 (6.5)	31.9
Saraya Co. Ltd	7 (4.5)	39.4
Agency For Science Technology Research (A*Star), Singapore	6 (3.9)	32.7
Tianjin Institute of Industrial Biotechnology, Chinese Academy of Sciences	6 (3.9)	22.2
University of Illinois System	6 (3.9)	40.3
**Country/region**		
China	67 (43.2)	18.4
United States	41 (26.5)	29.2
Japan	17 (11.0)	38.2
Brazil	6 (3.9)	9.0
Canada	6 (3.9)	13.7
Singapore	6 (3.9)	32.7
South Korea	6 (3.9)	14.0
**Journal**		
Journal of Agricultural and Food Chemistry	10 (6.5)	29.0
Food Chemistry	8 (5.2)	18.4
Molecules	7 (4.5)	10.0
Journal of Dairy Science	6 (3.9)	15.2
Journal of Food Science	6 (3.9)	11.8
**Journal category**		
Food Science Technology	67 (43.2)	15.6
Chemistry Applied	24 (15.5)	21.2
Nutrition Dietetics	23 (14.8)	31.2
Pharmacology Pharmacy	22 (14.2)	26.4
Biochemistry Molecular Biology	14 (9.0)	11.2

**Figure 2:**
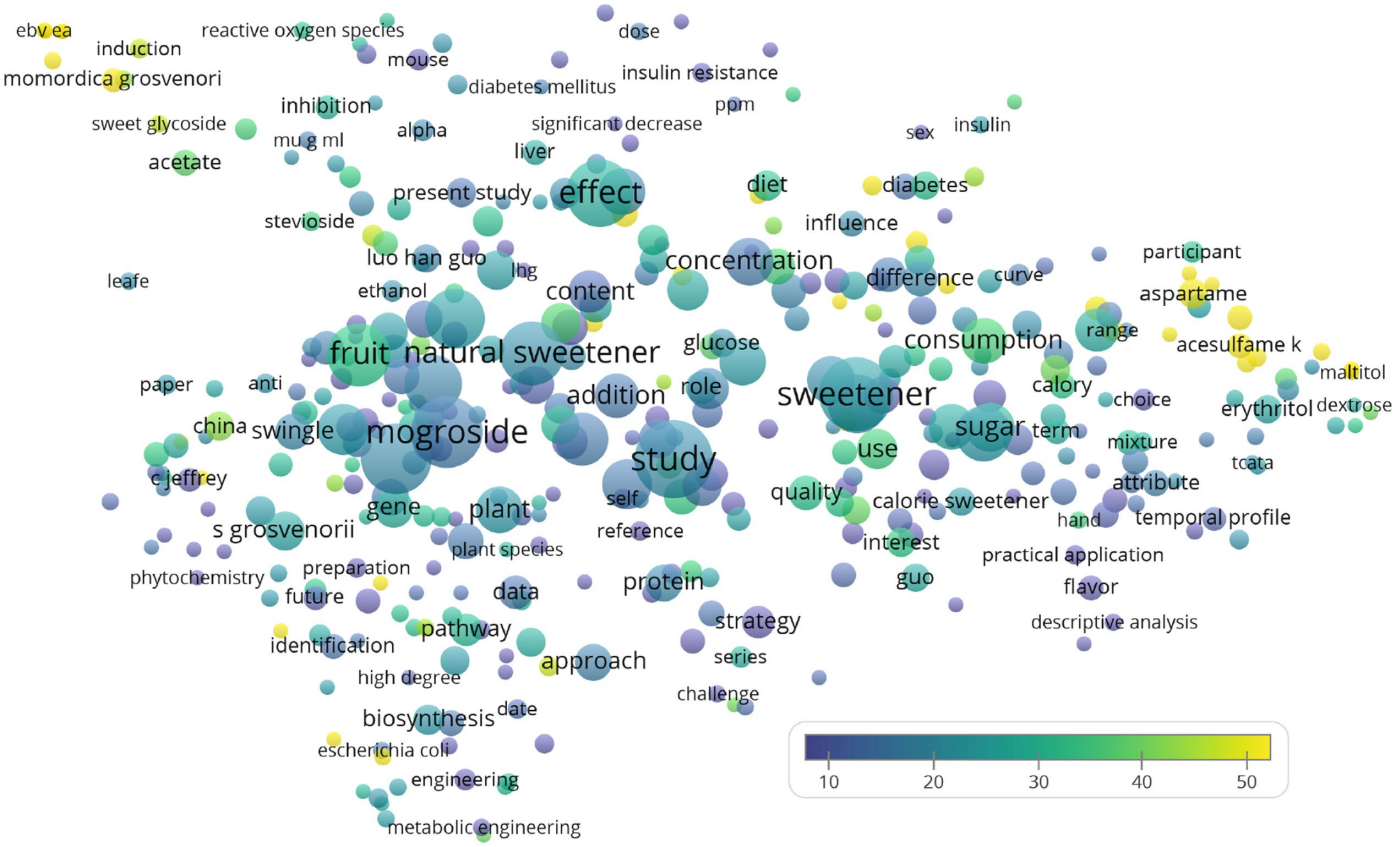
Term map showing the recurring terms in the title and abstracts of the analyzed literature.

The recurring terms in the title and abstracts of the analyzed literature are visualized in Figure 2. On the right side of the figure, it could be observed that papers that mentioned some low-calorie or non-nutritive sweeteners generally had a higher CPP (yellow nodes), such as aspartame (*n* = 11, CPP = 49.2), acesulfame K (*n* = 8, CPP = 59.9), and maltitol (*n* = 3, CPP = 72.3). Mogroside could be a helpful sweetener for patients suffering from diabetes (*n* = 10, CPP = 31.5).

The top 10 most cited papers are listed in Table 3. The health effects and biosynthesis of mogrosides were the recurring themes among these publications. Data from Scite indicated that most of the citations they received were mere mentioning in nature, without testing the reported results.

**Table 3:** The top 10 most cited papers of the analyzed literature.

Rank	Paper	Citation count from Web of Science	Citation context (supporting: mentioning: contrasting) from Scite
1	Position of the Academy of Nutrition and Dietetics: Use of Nutritive and Nonnutritive Sweeteners (21)	234	0:44:0
2	Trends in use, pharmacology, and clinical applications of emerging herbal nutraceuticals (22)	146	0:130:0
3	The role of artificial and natural sweeteners in reducing the consumption of table sugar: A narrative review (5)	136	1:134:0
4	An efficient approach to finding *Siraitia grosvenorii* triterpene biosynthetic genes by RNA-seq and digital gene expression analysis (23)	130	3:78:0
5	The biosynthetic pathway of the nonsugar, high-intensity sweetener mogroside V from *Siraitia grosvenorii* (24)	106	4:118:0
6	Anticarcinogenic activity of natural sweeteners, cucurbitane glycosides, from *Momordica grosvenori* (25)	105	0:69:0
7	Chemistry and pharmacology of *Siraitia grosvenorii*: A review (26)	98	0:6:0
8	Sweeteners from plants-with emphasis on *Stevia rebaudiana* (Bertoni) and *Siraitia grosvenorii* (Swingle) (27)	93	1:80:0
9	Anti-inflammatory Activities of Mogrosides from *Momordica grosvenori* in Murine Macrophages and a Murine Ear Edema Model (8)	76	0:33:0
10	Triterpene glycosides of *Siraitia grosvenori* inhibit rat intestinal maltase and suppress the rise in blood glucose level after a single oral administration of maltose in rats (28)	74	2:28:1

Web of Science and Scite counted citation number differently, and therefore the numbers of the last two columns did not match each other.

**Figure 3:**
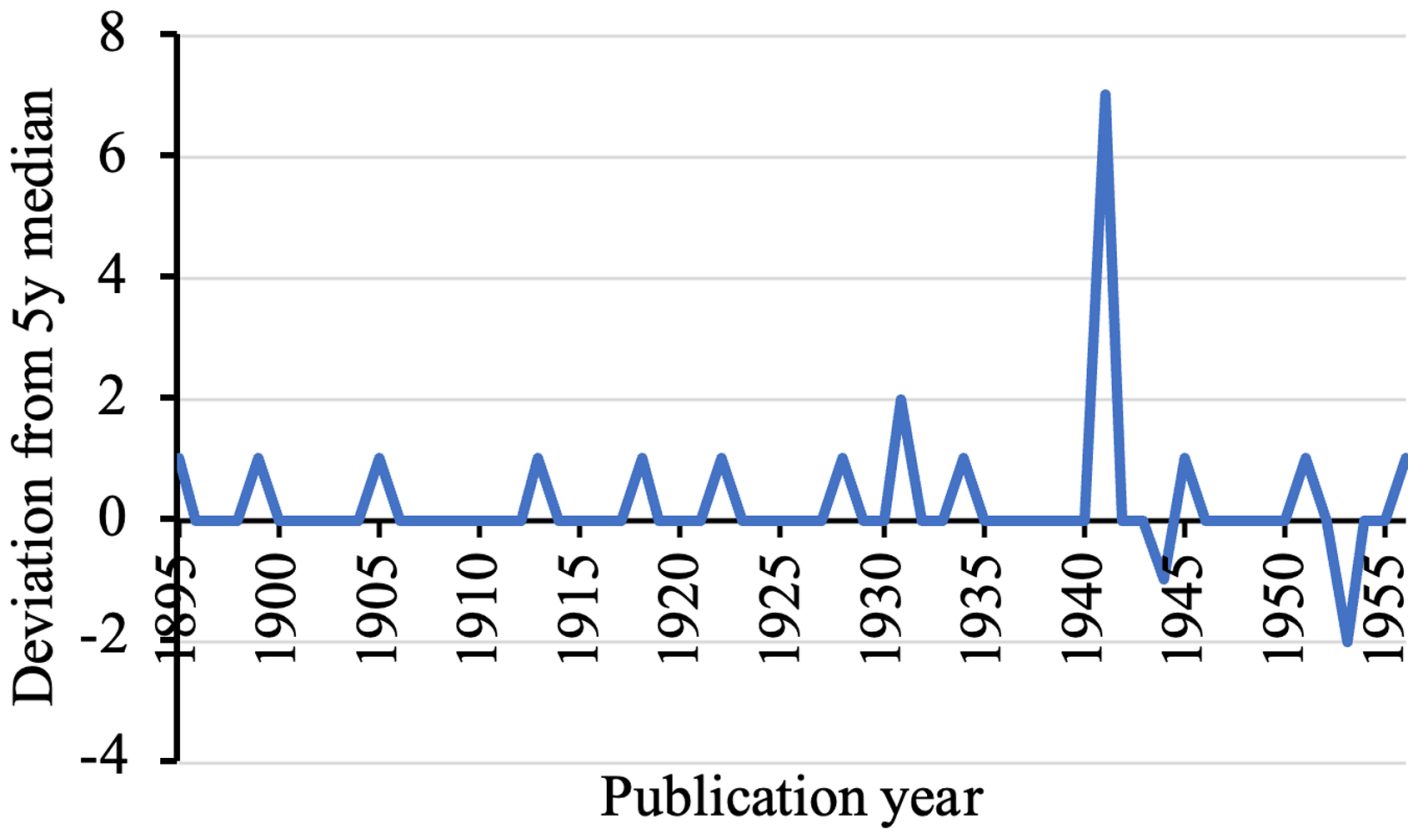
RPYS of the cited references published until 1956.

Finally, Figure 3 shows a RPYS of the cited references published until 1956. There was one obvious peak identified in 1941. Within this dataset, the cited references published in 1939, 1940, 1941, 1942, and 1943 were collectively cited 0, 0, 8, 1, and 1 times. The 5-year median was 1. Subsequently, the deviation from this median was 7 (8-1) in year 1941, producing a peak in the RPYS with a magnitude of 7. By examining the data, all 8 citations in year 1941 were made to Swingle (29). In this paper, the author, Walter Tennyson Swingle, described the appearance of the monk fruit plant and earlier botanical expeditions in China, and named the monk fruit *Momordica grosvenorii*, in honor of Dr. Gilbert Grosvenor, then-president of the National Geographic Society who funded the expeditions in China.

## Discussion

This bibliometric analysis on monk fruit extract and mogroside research analyzed 155 papers. Nearly half of them had contributions from China and were published in journals concerning food science technology. The health effects and biosynthesis of mogrosides were the recurring themes among the top 10 most cited publications. Papers mentioning other sweeteners or sugar alcohols received many citations in general.

Understanding the biosynthesis of mogrosides may help scientists to improve their productivity within monk fruit plant or even in other genetically modified plants. For instance, Tang et al. (23) (ranked 4th in Table 3) identified seven cytochrome P450 (CYP450) and five UDP-glucosyltransferases (UDPG) as potential candidates responsible for the biosynthesis of mogrosides. After that, Itkin et al. (24) (ranked 5th in Table 3) identified the members of five enzyme families responsible for mogroside biosynthesis, namely the squalene epoxidases, triterpenoid synthases, epoxide hydrolases, CYP450, and UDPG. Very recently, successful mogroside biosynthesis in transgenic cucumber and tomato (30) and transgenic *Nicotiana benthamiana* (benth) and *Arabidopsis thaliana* (thale cress) (31) have been reported.

Meanwhile, animal studies showed promising results on the health effects of monk fruit extract or mogrosides. A mouse study reported an inhibitory effects of mogroside V and 11-oxo-mogroside V on mouse skin carcinogenesis models (25) (ranked 6th in Table 3). Another mouse study found that mogrosides were able to down-regulate the expression of inflammatory genes and up-regulate inflammation protective genes both with the cell model (macrophage) and organ model (ear) (8) (ranked 9th in Table 3). A rat study showed that the increase in plasma glucose level in response to maltose intake was significantly suppressed by ingesting monk fruit extract 3 min before the maltose intake, and this suppression was not observed by replacing monk fruit extract with glucose (28) (ranked 10th in Table 3). The authors found that monk fruit extract inhibited the small intestinal maltase in the rat and hence exerted the observed anti-hyperglycemic effect. Further, mogrosides could also exhibit both anti-hyperglycemic and anti-hyperlipidemic effects on rats with type 2 diabetes, and regulate insulin secretion by increasing the glucagon-like peptide-1 (GLP-1) (32). Few clinical studies, if not none, have been conducted to test such health benefits in humans.

With such potential health benefits, monk fruit extract or mogrosides could be desirable sugar substitutes. However, the flavor profile of monk fruit extract is different from sucrose. For instance, while sucrose was characterized by a fast sweetness onset to peak intensity and a subsequent decay with minimal side tastes, monk fruit extract had prominent and long-lasting intensities of the undesirable bitter, metallic and chemical side tastes comparable to other non-nutritive sweeteners (33). The metallic taste could be significantly reduced (though together with sweet taste) via transglycosylation (34). Non-nutritive sweeteners could be mixed/blended to produce a temporal sensory profile that matched that of sucrose more closely, and label-conscious consumers would prefer beverages sweetened by natural sweeteners (such as monk fruit extract and stevia) over artificial sweetener (e.g., sucralose) (11).

This bibliometric study had some limitations. For example, WoS Core Collection became more comprehensive in indexing the abstract, author keywords, and keywords plus information after 1991 (35), so that the “hit rate” of any search not limited to the title field would be lower before 1991 (36). Readers should also note that this study relied on a single database with a customized subscription. Publications not indexed by it would be omitted in the analysis. For instance, some publications indexed in Scopus and some preprints would be omitted. However, this study also had some strengths. In particular, the use of cited reference analysis in the form of RPYS ultimately identified a commonly cited reference published back in 1941, which was beyond the timespan of the literature set subscribed by the author’s affiliation. This particular reference by Swingle (29) is very crucial and has a historical significance, as it gave the first scientific name to monk fruit. According to WoS “cited reference search,” this reference received 12 citations only. Even if the author could search with a longer timespan, this paper would not stand out through the usual citation analysis.

Lots of animal studies, particularly those with rat models, have demonstrated many potential health benefits of monk fruit extracts and mogrosides. For future perspectives, more clinical trials should be performed. Regarding their roles as non-nutritive sweeteners, more studies should be performed to evaluate the optimal amount/concentration to be added into food items and beverages to replace sugars without altering the sweetness profile too much.

To conclude, nearly half of the publications on monk fruit extract and mogrosides had contributions from China and were published in journals concerning food science technology. The health effects and biosynthesis of mogrosides were the recurring themes among the top 10 most cited publications. Papers mentioning other sweeteners or sugar alcohols received many citations in general. More future studies should be conducted to test the various health benefits in humans. Also, one future research trend could be mass scale production of mogrosides from transgenic crops that can be cultivated more easily across the globe.

## Data availability statement

The original contributions presented in the study are included in the article/supplementary material, further inquiries can be directed to the corresponding author.

## Author contributions

The author confirms being the sole contributor of this work and has approved it for publication.

## Funding

This work was supported by departmental funds only.

## Conflict of interest

The author declares that the research was conducted in the absence of any commercial or financial relationships that could be construed as a potential conflict of interest.

## Publisher’s note

All claims expressed in this article are solely those of the authors and do not necessarily represent those of their affiliated organizations, or those of the publisher, the editors and the reviewers. Any product that may be evaluated in this article, or claim that may be made by its manufacturer, is not guaranteed or endorsed by the publisher.

## References

[1] Soejarto DD Addo EM Kinghorn AD. Highly sweet compounds of plant origin: from ethnobotanical observations to wide utilization. J Ethnopharmacol. (2019) 243:112056. doi: 10.1016/j.jep.2019.112056, PMID: 31279071

[2] Xie B Lai B Chen L Wei S Tang S. Phylogeographic analysis of Siraitia grosvenorii in subtropical China provides insights into the origin of cultivated monk fruit and conservation of genetic resources. Ecol Evol. (2023) 13:e10181. doi: 10.1002/ece3.10181, PMID: 37304364 PMC10256620

[3] Salanță LC Uifălean A Iuga C-A Tofană M Cropotova J Pop OL . Valuable food molecules with potential benefits for human health. In The health benefits of foods-current knowledge and further development [internet]. London: IntechOpen (2020). 1–45.

[4] Wang B Yang Z Xin Z Ma G Qian Y Xie T . Analysis of Mogrosides in Siraitia Grosvenorii fruits at different stages of maturity. Nat Prod Commun. (2019) 14:1934578X1987862. doi: 10.1177/1934578X19878621

[5] Mooradian AD Smith M Tokuda M. The role of artificial and natural sweeteners in reducing the consumption of table sugar: a narrative review. Clin Nutr ESPEN. (2017) 18:1–8. doi: 10.1016/j.clnesp.2017.01.004, PMID: 29132732

[6] Liu X Zhang J Li Y Sun L Xiao Y Gao W . Mogroside derivatives exert hypoglycemics effects by decreasing blood glucose level in Hepg2 cells and alleviates insulin resistance in T2dm rats. J Funct Foods. (2019) 63:103566. doi: 10.1016/j.jff.2019.103566

[7] Liu H Wang C Qi X Zou J Sun Z. Antiglycation and antioxidant activities of Mogroside extract from Siraitia Grosvenorii (Swingle) fruits. J Food Sci Technol. (2018) 55:1880–8. doi: 10.1007/s13197-018-3105-2, PMID: 29666541 PMC5897311

[8] Di R Huang M-T Ho C-T. Anti-inflammatory activities of Mogrosides from *Momordica grosvenori* in murine macrophages and a murine ear edema model. J Agric Food Chem. (2011) 59:7474–81. doi: 10.1021/jf201207m 21631112

[9] Chadha D Hamid N Kantono K Marsan M. Changes in temporal sensory profile, liking, satiety, and Postconsumption attributes of yogurt with natural sweeteners. J Food Sci. (2022) 87:3190–206. doi: 10.1111/1750-3841.16224, PMID: 35708195 PMC9545239

[10] Li X Lopetcharat K Drake M. Parents’ and Children's acceptance of skim chocolate milks sweetened by monk fruit and stevia leaf extracts. J Food Sci. (2015) 80:S1083–92. doi: 10.1111/1750-3841.12835, PMID: 25847181

[11] Parker M Lopetcharat K Drake M. Consumer acceptance of natural sweeteners in protein beverages. J Dairy Sci. (2018) 101:8875–89. doi: 10.3168/jds.2018-14707, PMID: 30055918

[12] van Eck NJ Waltman L. Software survey: Vosviewer, a computer program for bibliometric mapping. Scientometrics. (2010) 84:523–38. doi: 10.1007/s11192-009-0146-3, PMID: 20585380 PMC2883932

[13] Nicholson JM Mordaunt M Lopez P Uppala A Rosati D Rodrigues NP . Scite: a smart citation index that displays the context of citations and classifies their intent using deep learning. Quant Sci Stud. (2021) 2:882–98. doi: 10.1162/qss_a_00146

[14] Thor A Marx W Leydesdorff L Bornmann L. Introducing Citedreferencesexplorer (Crexplorer): a program for reference publication year spectroscopy with cited references standardization. J Informetr. (2016) 10:503–15. doi: 10.1016/j.joi.2016.02.005

[15] Marx W Bornmann L. Tracing the origin of a scientific legend by reference publication year spectroscopy (Rpys): the legend of the Darwin finches. Scientometrics. (2014) 99:839–44. doi: 10.1007/s11192-013-1200-8

[16] Marx W Bornmann L Barth A Leydesdorff L. Detecting the historical roots of research fields by reference publication year spectroscopy (Rpys). J Assoc Inf Sci Technol. (2014) 65:751–64. doi: 10.1002/asi.23089

[17] Wray KB Bornmann L. Philosophy of science viewed through the Lense of “referenced publication years spectroscopy”(Rpys). Scientometrics. (2015) 102:1987–96. doi: 10.1007/s11192-014-1465-6

[18] Yeung AWK. Identification of seminal works that built the Foundation for Functional Magnetic Resonance Imaging Studies of taste and food. Curr Sci. (2017) 113:1225–7.

[19] Yeung AWK. A bibliometric analysis on the early works of dental anxiety. Dent J. (2023) 11:36. doi: 10.3390/dj11020036, PMID: 36826181 PMC9955892

[20] Yeung AWK Wong NSM. The historical roots of visual analog scale in psychology as revealed by reference publication year spectroscopy. Front Hum Neurosci. (2019) 13:86. doi: 10.3389/fnhum.2019.00086, PMID: 30914939 PMC6423150

[21] Fitch C Keim KS. Position of the academy of nutrition and dietetics: use of nutritive and nonnutritive sweeteners. J Acad Nutr Diet. (2012) 112:739–58. doi: 10.1016/j.jand.2012.03.009, PMID: 22709780

[22] Williamson EM Liu X Izzo AA. Trends in use, pharmacology, and clinical applications of emerging herbal nutraceuticals. Br J Pharmacol. (2020) 177:1227–40. doi: 10.1111/bph.14943, PMID: 31799702 PMC7056462

[23] Tang Q Ma X Mo C Wilson IW Song C Zhao H . An efficient approach to finding Siraitia Grosvenorii triterpene biosynthetic genes by Rna-seq and digital gene expression analysis. BMC Genomics. (2011) 12:343. doi: 10.1186/1471-2164-12-343, PMID: 21729270 PMC3161973

[24] Itkin M Davidovich-Rikanati R Cohen S Portnoy V Doron-Faigenboim A Oren E . The biosynthetic pathway of the nonsugar, high-intensity sweetener Mogroside V from Siraitia Grosvenorii. Proc Natl Acad Sci. (2016) 113:E7619–28. doi: 10.1073/pnas.1604828113 27821754 PMC5127336

[25] Takasaki M Konoshima T Murata Y Sugiura M Nishino H Tokuda H . Anticarcinogenic activity of natural sweeteners, Cucurbitane glycosides, from *Momordica grosvenori*. Cancer Lett. (2003) 198:37–42. doi: 10.1016/S0304-3835(03)00285-4, PMID: 12893428

[26] Chun L Li-Mei L Feng S Zhi-Min W Hai-Ru H Li D . Chemistry and pharmacology of Siraitia Grosvenorii: a review. Chin J Nat Med. (2014) 12:89–102. doi: 10.1016/S1875-5364(14)60015-7 24636058

[27] Pawar RS Krynitsky AJ Rader JI. Sweeteners from plants—with emphasis on *Stevia Rebaudiana* (Bertoni) and Siraitia Grosvenorii (Swingle). Anal Bioanal Chem. (2013) 405:4397–407. doi: 10.1007/s00216-012-6693-0, PMID: 23341001

[28] Suzuki YA Murata Y Inui H Sugiura M Nakano Y. Triterpene glycosides of Siraitia Grosvenori inhibit rat intestinal maltase and suppress the rise in blood glucose level after a single Oral Administration of Maltose in rats. J Agric Food Chem. (2005) 53:2941–6. doi: 10.1021/jf0478105 15826043

[29] Swingle WT. *Momordica grosvenori* Sp. Nov. the source of the Chinese lo Han Kuo. J Arnold Arboretum. (1941) 22:197–203. doi: 10.5962/p.183529

[30] Liao J Liu T Xie L Mo C Qiao J Huang X . Heterologous Mogrosides biosynthesis in cucumber and tomato by genetic manipulation. Commun Biol. (2023) 6:191. doi: 10.1038/s42003-023-04553-3, PMID: 36805532 PMC9938114

[31] Liao J Liu T Xie L Mo C Huang X Cui S . Plant metabolic engineering by multigene stacking: synthesis of diverse Mogrosides. Int J Mol Sci. (2022) 23:10422. doi: 10.3390/ijms231810422 36142335 PMC9499096

[32] Zhang Y Zhou G Peng Y Wang M Li X. Anti-hyperglycemic and anti-hyperlipidemic effects of a special fraction of Luohanguo extract on obese T2dm rats. J Ethnopharmacol. (2020) 247:112273. doi: 10.1016/j.jep.2019.112273, PMID: 31586692

[33] Tan VWK Wee MSM Tomic O Forde CG. Temporal sweetness and side tastes profiles of 16 sweeteners using temporal check-all-that-apply (Tcata). Food Res Int. (2019) 121:39–47. doi: 10.1016/j.foodres.2019.03.019, PMID: 31108762

[34] Muñoz-Labrador A Azcarate S Lebrón-Aguilar R Quintanilla-López JE Galindo-Iranzo P Kolida S . High-yield synthesis of Transglycosylated Mogrosides improves the flavor profile of monk fruit extract sweeteners. J Agric Food Chem. (2021) 69:1011–9. doi: 10.1021/acs.jafc.0c07267, PMID: 33428404

[35] Liu F. Retrieval strategy and possible explanations for the abnormal growth of research publications: re-evaluating a bibliometric analysis of climate change. Scientometrics. (2023) 128:853–9. doi: 10.1007/s11192-022-04540-1, PMID: 36274793 PMC9574164

[36] Liu W. Caveats for the use of web of science Core collection in old literature retrieval and historical bibliometric analysis. Technol Forecast Soc Chang. (2021) 172:121023. doi: 10.1016/j.techfore.2021.121023

